# Time-Varying Gene Network Analysis of Human Prefrontal Cortex Development

**DOI:** 10.3389/fgene.2020.574543

**Published:** 2020-11-16

**Authors:** Huihui Wang, Yongqing Wu, Ruiling Fang, Jian Sa, Zhi Li, Hongyan Cao, Yuehua Cui

**Affiliations:** ^1^Division of Health Statistics, School of Public Health, Shanxi Medical University, Taiyuan, China; ^2^Department of Hematology, Taiyuan Central Hospital of Shanxi Medical University, Taiyuan, China; ^3^Department of Statistics and Probability, Michigan State University, East Lansing, MI, United States

**Keywords:** human PFC, gene network change, time-varying graph, loggle model, hub gene

## Abstract

The prefrontal cortex (PFC) constitutes a large part of the human central nervous system and is essential for the normal social affection and executive function of humans and other primates. Despite ongoing research in this region, the development of interactions between PFC genes over the lifespan is still unknown. To investigate the conversion of PFC gene interaction networks and further identify hub genes, we obtained time-series gene expression data of human PFC tissues from the Gene Expression Omnibus (GEO) database. A statistical model, loggle, was used to construct time-varying networks and several common network attributes were used to explore the development of PFC gene networks with age. Network similarity analysis showed that the development of human PFC is divided into three stages, namely, fast development period, deceleration to stationary period, and recession period. We identified some genes related to PFC development at these different stages, including genes involved in neuronal differentiation or synapse formation, genes involved in nerve impulse transmission, and genes involved in the development of myelin around neurons. Some of these genes are consistent with findings in previous reports. At the same time, we explored the development of several known KEGG pathways in PFC and corresponding hub genes. This study clarified the development trajectory of the interaction between PFC genes, and proposed a set of candidate genes related to PFC development, which helps further study of human brain development at the genomic level supplemental to regular anatomical analyses. The analytical process used in this study, involving the loggle model, similarity analysis, and central analysis, provides a comprehensive strategy to gain novel insights into the evolution and development of brain networks in other organisms.

## Introduction

The prefrontal cortex (PFC), covering the front part of the frontal lobe, receives input from multiple regions of the brain for information processing (Fuster, 2001; Hathaway and Newton, 2019), and is a key area for studying the development and mechanisms of decline of the human brain. It plays important roles in emotional and social behavior, coordinating complex cognitive behavior, expression, and decision-making (Fuster, 2003; Fellows, 2007). PFC development is greatly shaped by gene expression, which is dynamically regulated across a person’s lifespan (Jaffe et al., 2014). By mapping the key features of the developmental trajectory of PFC gene expression, not only can the dynamic development of brain function be revealed, but also our understanding of the mechanisms that drive cellular responses can be promoted (Shaw et al., 2008; Molnár et al., 2019).

Since it is obviously impossible to perform biopsies from the same area of an individual’s brain multiple times during growth to generate time-series genetic data, in the past few decades, researchers have evaluated the developmental trajectory of the forehead from the perspectives of neuropsychology, neuroimaging, and cell physiology (Diamond and Doar, 1989; Case, 1992; Luciana and Nelson, 1998; Anderson et al., 2001). It is generally believed that the neuro-physiological development of the forehead experiences a pattern of first increasing and then decreasing to a steady state. For example, Shaw et al. (2008) simulated the changes in the cerebral cortex by combining longitudinal neuroanatomical imaging data with cross-sectional data. They found that the developmental trajectory of the frontal cortex was cubic, that is, it increases first and then gradually decreases to a steady state (Shaw et al., 2008). With the advancement of science and technology, researchers have constructed time-series genetic data from a single biopsy of multiple individuals and characterized similar developmental trajectories at the genetic level, but only explored this based on the expression level of individual genes (Kolb et al., 2012; Wruck and Adjaye, 2020). For example, Liu et al. (2012) used unsupervised hierarchical clustering to cluster the PFC genes and found that the expression levels of genes related to neuronal activity show a trend of rising then decreasing throughout the lifespan. Although previous studies clearly observed age-related changes in PFC development from the anatomical structure and individual gene expression levels, the generation of cell diversity during human brain development requires precise regulation between genes (Moreau et al., 2013; Wang and Wang, 2019). The temporal dynamics of this intergenic interaction is yet to be delineated.

As a statistical tool, network analysis can help us fully understand the internal complex systems, rather than just individual genes functioning along (Chandrasekaran and Bonchev, 2016). However, network graphs created with time-varying data may change over time. If simply integrating a static network at different time points for dynamic analysis (Faisal and Milenković, 2014), one may not make full use of the advantages of time series data, hence may not be able to capture the complex dynamic biological phenomena on the time axis (Androulakis et al., 2007). Moreover, the integration of time series data will bring a large calculation burden (Zhang et al., 2010; Yang and Peng, 2018). To cope with the challenge of time-series data, some methods have been developed based on the Gaussian Graphic Model (GGM) (Drton and Perlman, 2004) to estimate time-varying graphs (Le et al., 2009; Kolar and Xing, 2013; Gibberd and Nelson, 2014, 2015) while assuming that the covariance matrices change smoothly over time; this facilitates understanding and explanation of the interaction of network nodes. Among them, the Local Group Graphical Lasso Estimation (loggle) (Yang and Peng, 2018) model proposed by Yang and Peng, not only effectively uses the neighborhood information by using a local group-lasso-type penalty, but also saves computational time by using a blockwise fast algorithm and pseudo-likelihood approximation. It has advantages over other time-varying graph models using fused-lasso-type penalties which estimate the piecewise constant to identify the jump points [e.g., TESLA (Ahmed and Xing, 2009), TVGL (Hallac et al., 2017), GFGL (Gibberd and Nelson, 2017)]. The successful application of the loggle model in the work of Yang and Peng (2018) illustrates how direct interactions between stocks evolved over time under the influence of the global financial crisis.

In this work, we apply the loggle model to a time-series gene expression data set to construct PFC time-varying gene interaction networks, since the model fits the biological realm of PFC development. We quantify the development trend of the PFC gene network through network global attribute indicators such as network diameter. We further apply network similarity analysis to describe the development stage of PFC, so as to identify hub genes at different stages using the central analysis. We also apply the loggle model to evaluate the development of several KEGG pathways in PFC. The identification of the changes of gene networks in human PFC can provide novel insights into human brain development and function. The hub genes identified in different development stages provide specific candidate targets for further biological validation.

## Materials and Methods

### Data

#### Human PFC Time-Series Gene Expression Data

The time-series gene expression data on the human PFC were downloaded from the GEO database^1^ with Gene Expression Omnibus accession number GSE30272. This data set records 269 RNA samples from stages from fetus development to elderly (14 gestational weeks to 80 years), after removing subjects with severe neurological or psychiatric conditions. These samples were obtained from post-mortem human brain PFC gray matter tissue homogenates and subjected to a series of processes such as RNA extraction and quality control. The log2 intensity ratio was normalized after background correction, and the log2 ratio was further adjusted to reduce the impact of systematic noise after performing surrogate variable analysis. Readers are referred to the paper by Colantuoni et al. (2011) for a detailed description of the data source and processing procedures. After probe annotation and data cleaning, a time-series gene expression matrix of 17,150 × 269 was generated for further statistical analysis.

#### Initial Feature Selection

Considering data noise and the complexity of the algorithms that would be used to construct a time-varying graph, we first performed feature screening to filter out potential noise and reduce the data dimensionality. In this work, we considered the following two options for feature screening to obtain genes that carry important information.

(i) Calculate the variance for each gene and select the top 300 genes to construct the time-varying network graphs. The purpose of this is to explore the development of networks constructed with genes showing high variation in PFC throughout the lifespan, or changes in the interactions between the dominant genes at different developmental stages of PFC.

(ii) Select genes based on known KEGG pathways. The purpose of this is to explore the development trends of several known pathways in the PFC throughout the lifespan. In this study, we chose five pathways related to the development of PFC function by searching the literature. A list of the pathways is shown in Table 1, together with the pathway entry and name, the number of genes in the pathway, and the number of genes mapped to the pathway in the original gene expression data set. A total of five systems related to PFC or sensitive to age changes are involved, namely, signal transduction (hsa04068), immune system (hsa04611), nervous system (hsa04728), aging (hsa04211), and development and regeneration (hsa04360).

**TABLE 1:** Selected pathway information.

Pathway entry	Pathway name	# of genes in the pathway	# of genes mapped to the pathway
hsa04728	*Dopaminergic synapse pathway*	131	123
hsa04211	*Longevity regulating pathway*	89	80
hsa04360	*Axon guidance pathway*	181	171
hsa04611	*Platelet activation pathway*	124	110
hsa04068	*FoxO signaling pathway*	131	118

#### Age Grouping

**FIGURE 1:**
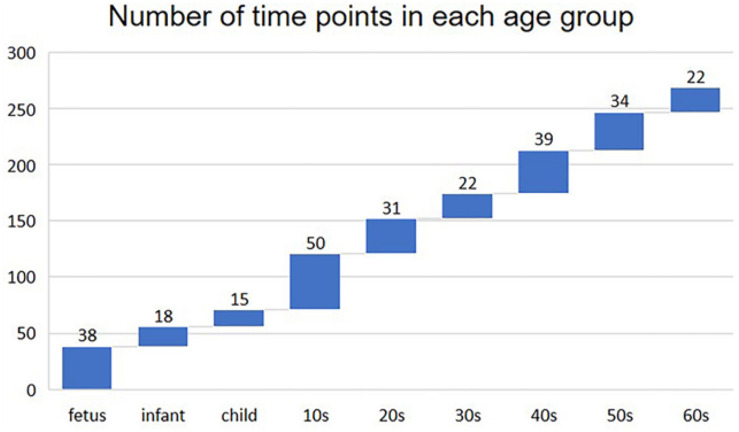
Number of time points (samples) in each age group.

We built the time-varying network by dividing the sample into nine age periods based on the age information provided by the original data (Colantuoni et al., 2011), that is, fetus (14–20 gestational weeks), infant (0–6 months), child (1–10 years), 10s (10–20 years), 20s (20–30 years), 30s (30–40 years), 40s (40–50 years), 50s (50–60 years), and 60s (60 years or older). The distribution of the number of time points (samples) in each age group is shown in Figure 1.

### Estimating Time-Varying Graphs With the Loggle Model

This study aims to characterize the developmental pattern of inter-gene interactions over time in the human PFC region and identify hub genes involved in development. Accordingly, we first used the loggle model to build and understand PFC time-varying network graphs. In particular, the loggle model uses the local group-lasso penalty to minimize locally weighted negative log-likelihood function to reasonably combine the information of adjacent time points to ensure the progressive change of the graph structure. Then, a blockwise fast algorithm and pseudo-likelihood approximation were used to solve the “computational disaster” problem. The PFC time-varying graphs were constructed using the loggle package in R. To make the work self-contained, we here briefly describe how to construct the time-varying network graph via the loggle model. More technical details can be found in the paper by Yang and Peng (2018).

#### Local Group Graphical Lasso Estimation

Suppose X(t) = (*X*^1^(t),*X*^2^(t),…,*X*^*p*^(t))^*T*^ is a *p*-dimensionalg time-series random vector at time *t*∈[0, 1], which obeys a multivariate Gaussian distribution 𝒩_*p*_(μ(*t*),∑(*t*)). We used {*x*_*k*_} (*k*∈{1, …, N}) to indicate the observation at time *t_k_*(0 = *t*_1_ = … = *t_k_* = … *t_N_* = 1), where *N* represents the sample size. For simplicity, we centered the observations *x_k_* by subtracting the estimated mean $\hat{\mu }\left(t_{k}\right)$ from *x_k_* so that each *x_k_*is drawn independently from 𝒩_*p*_(0,∑(*t*)).

We next estimated the precision matrix Ω(*t*)⋅(Ω(*t*) = Σ^−1^(*t*)) to construct the graph edge set. The loggle model assumes the smoothness of the graphical topology, and obtains the estimated precision matrix $\hat{\Omega }\left(t\right)$ at the *k*th time point by combining the locally weighted negative log-likelihood function with the local group lasso penalty (Ming and Yi, 2006):

(1) $$\begin{array}{c} L\left(\Omega_{k}\right):=\frac{1}{\sqrt{\left|N_{k,d}\right|}}\Sigma_{i\in N_{k,d}}\left[tr\left(\Omega \left(t_{i}\right)\hat{\Sigma }\left(t_{i}\right)\right)-\log\left|\Omega \left(t_{i}\right)\right|\right]\\ +\lambda \Sigma_{u\neq v}\sqrt{\Sigma_{i\in N_{k,d}}\Omega_{uv}{\left(t_{i}\right)}^{2}}, \end{array}$$

where *N*_*k*,*d*_ = {*i* ∈ *I*:|*t*_*i*_−*t*_*k*_|≤*d*} is the time index with the center *t*_*k*_ and neighborhood width *d*; |*N*_*k*,*d*_| is the cardinality of *N*_*k,d*_; Ω_*k*_ = {Ω(*t*_*i*_)}_*i* ∈ *N*_*k*,*d*__ is a set of precision matrices with Ω_*uv*_(*t*_*i*_) representing the (*u, v*)-th element in Ω; $\hat{\Sigma }\left(t_{i}\right)=\Sigma_{j=1}^{N}\omega_{h}^{t_{j}}\left(t\right)x_{j}x_{j}^{T}$ is the kernel estimate of the covariance matrix, with $\omega_{h}^{t_{j}}\left(t\right)=\frac{k_{h}\left(t_{j}-t\right)}{\Sigma_{j=1}^{N}k_{h}\left(t_{j}-t\right)}$ as the weight and *K*_*h*_(⋅) = *K*(⋅/h) as a symmetric non-negative kernel function with bandwidth *h*.

#### Model Fitting and Optimization

The model uses the alternating directions method of multipliers (ADMM) algorithm (Boyd et al., 2010) to solve the convex optimization problem for objective function (1). Unfortunately, the ADMM algorithm involves eigen-decomposition, which can take a long time when the data dimensionality is large. To solve the “computational disaster” problem, the algorithm introduces a fast blockwise algorithm (Witten et al., 2011; Danaher et al., 2014) and a pseudo-likelihood approximation (Meinshausen and Bühlmann, 2006; Peng et al., 2009a, b) to the objective function.

Specifically, the *p* variables are completely separated into multiple non-overlapping blocks by the following necessary and sufficient condition after suitable permutation; then, the ADMM algorithm is applied to each block to speed up the computation and reduce the calculation time from O(*p*^3^) to $\Sigma_{l=1}^{L}\text{O}\left(p_{l}^{3}\right)$. In addition, the pseudo-likelihood approximation can speed up the calculation efficiency by changing the problem of estimating the sparse pattern of the precision matrix to estimating the sparsity pattern of the regression coefficients. Further, the paired group lasso penalty (Friedman et al., 2010) is used to ensure the symmetry of the edge selection.

#### Parameter Adjustment

When learning the loggle model, there are three parameters involved: the kernel bandwidth *h*; the neighborhood width *d*, which controls the smoothness of the graph over time; and the sparsity parameter *λ*, which controls the degree of graph sparsity. The tuning parameters are learned by cross-validation (CV) at each age period. For this purpose, data are divided into training and validation sets. The CV score on the *j*th validation set at time *t*_*k*_ is defined as:

(2) $$\begin{array}{c} CV_{j}\left(t_{k};\lambda_{k},d_{k},h\right)=tr\left({\hat{\Omega }}_{-\left(j\right)}^{rf}\left(t_{k};d_{k},\lambda_{k},h\right){\hat{\Sigma }}_{\left(j\right)}\left(t_{k}\right)\right)\\ \;-\log\left|{\hat{\Omega }}_{-\left(j\right)}^{rf}\left(t_{k};d_{k},\lambda_{k},h\right)\right| \end{array}$$

**FIGURE 2:**
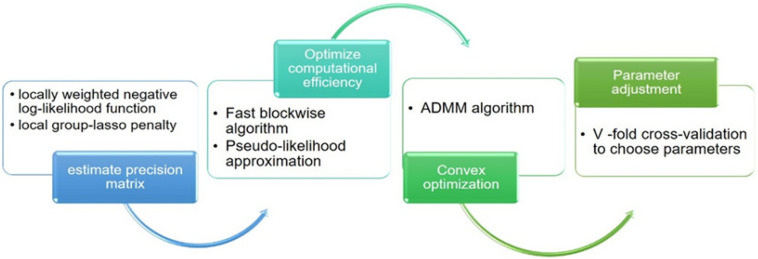
The main algorithm flow chart involved in the loggle model.

The *K*-fold CV score at time *t_k_* is defined as $\text{CV}\left(t_{k};;\lambda_{k},d_{k},h\right)=\sum_{j=1}^{K}CV_{j}\left(t_{k};;\lambda_{k},d_{k},h\right)$. The smallest CV score corresponds to the optimal combination of parameters (*h*, *λ_*k*_*, *d*_*k*_). At the same time, the “majority vote” procedure cv.vote (Peng et al., 2009b) was introduced to effectively reduce the false discovery rate. The algorithm flow involved in the loggle model is shown in Figure 2.

### Parameter Setting

Given the nine time points, we performed a threefold CV to determine the tuning parameters *h*, *d*, and *λ* of the graph at each time point. We initialize the range of h, d, and λ. Let *dat*_*j*_ (*j* = 1,…9) index the data in the *j*th time point. Each time, we set one of the three sets in (*dat*_1_, *dat*_4,_ *dat*_7_), (*dat*_2,_ *dat*_5,_ *dat*_8_), and (*dat*_3,_ *dat*_6,_ *dat*_9_) as the validation set, and the rest as the training set. The training set is used to estimate the correlation matrix of the graph model, and the validation set is used to calculate the cross-validation score. For a given *h*, we estimate Ω(*t*_*k*_;λ_*k*_,*d*_*k*_,*h*) based on the training set and calculate CV-score with the validation set. The loggle model assumes that data measured at different time points are independent and observations are made on a temporal grid, which makes the CV setting in this application valid (Yang and Peng, 2018). The early grid search stop threshold is 8; that is, the grid search stops when the number of edges exceeds 8*p* where *p* is the number of variables. The threshold for cv.vote is set to 0.8; that is, by fitting the model in each training set, only the edges that appear in at least 80% of these models are retained.

### Comparison With Other Models

We further compared the performance of loggle with two existing special models of loggle, namely, kernel and invar. kernel introduces a kernel estimate $\hat{\Sigma }\left(t\right)$ into the likelihood function (Zhou et al., 2010) and by setting the parameter *d* = 0, but the model ignores the potential smoothness of the graph. invar is performed to estimate Ω(*t*_*k*_) by using the global group lasso penalty (Wang and Kolar, 2014) with *d* = 1; thus, the generated graphs do not change across the entire time domain.

### Global Network Properties

Observing different network properties can provide valuable insights into the redistribution of genes within biological networks as well as the evolution of biological network structures. We used several common network properties to explain the trend of change of the network topology: number of nodes, number of edges, network *diameter*, network *density*, and *exclusive edges*.

The network *diameter* represents the maximal distance (shortest path) among all of the distances calculated between each pair of nodes in a network (Scardoni and Laudanna, 2012), that is, *D* = *max*_*i*,*j*_⁡δ_*min*_(*i*,*j*), where δ_*min*_(*i*,*j*) represents the shortest path between nodes *i* and *j*. A “small” network diameter indicates that nodes in the network are closely connected and the graph is compact. In particular, comparing network diameters at different time points can predict network development in a timely manner (Scardoni and Laudanna, 2012).

The *network density* shows the sparseness or density of the graph based on the number of connections per node set, and is defined as *d(G)* = $\frac{2\left|E\right|}{\left|V\right|\left(\left|V\right|-1\right)}$. The *exclusive edge* metric indicates that some edges belong to a certain network and do not appear in the rest of the network (Kuntal et al., 2016).

### Network Similarity Analysis

Considering that nine different time points will likely generate different networks, we calculated the similarity between networks and merged similar networks into groups. Based on this, we analyzed the development of PFC at different stages. This analysis used the *CompNet neighbor similarity index* (CNSI) to measure the similarity between two compared networks. CNSI measures the similarity of each pair of nodes by comparing the degree of overlap between the first neighbors of the nodes between two networks. A cumulative overall similarity score for all nodes is calculated to specify similarities between two compared networks (Kuntal et al., 2016), that is, $CNSI=\Sigma_{i=1}^{N}\frac{f_{n_{i}}^{A}\cap f_{n_{i}}^{B}}{f_{n_{i}}^{A}\cup f_{n_{i}}^{B}}$, where *n*_*i*_ represents the *i*-th node of the two compared networks, and $f_{n_{i}}^{A}$ and $f_{n_{i}}^{B}$ refer to the first neighbor of the *i*-th node in the corresponding two compared networks.

### Central Analysis

**FIGURE 3:**
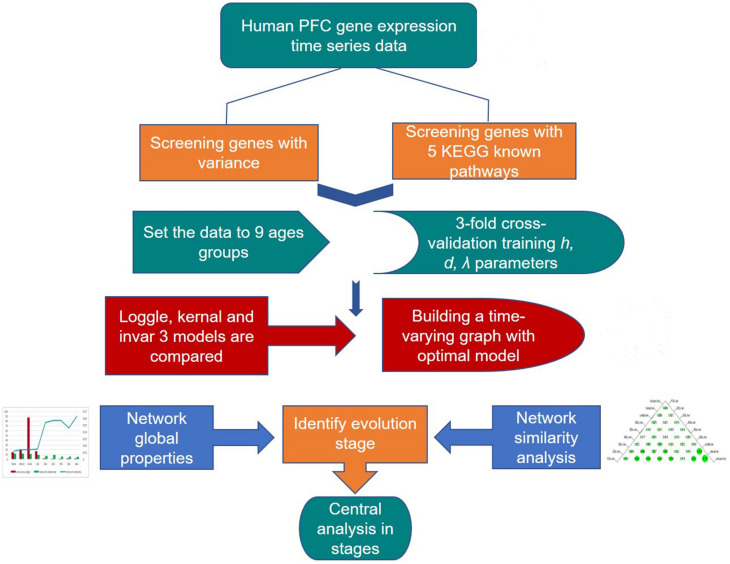
Detailed procedure for investigating the developmental pattern of human PFC gene interaction networks and hub genes.

Many known biological networks, such as signaling network, contain very few high-degree nodes but many low-degree ones, and nodes with very high degree play a central regulatory role (such as TP53) (Evans et al., 2019). Changes in some important nodes not only affect nodes adjacent to them, but also affect the topology of the entire network (Scardoni and Laudanna, 2012). To find important genes in human PFC tissue, we further calculated the *degree* of nodes to perform a central analysis. *Degree*, corresponding to the number of nodes directly connected to a given node *V* (the number of directly connected edges), namely, the first neighbors (Scardoni and Laudanna, 2012), is expressed as *C_*d*_(i)* = *deg(i)*. A high-degree node is called a “hub,” and removing such a node affects the network topology and further leads to disturbances in biological systems (Pavlopoulos et al., 2011). The degree calculation was performed in Cytoscape and is displayed with the node size corresponding to its value. Figure 3 shows a flow chart of the main methods and processes used for exploring the development of PFC in this study.

## Results

### Comparisons of Loggle, Kernel, and Invar

**FIGURE 4:**
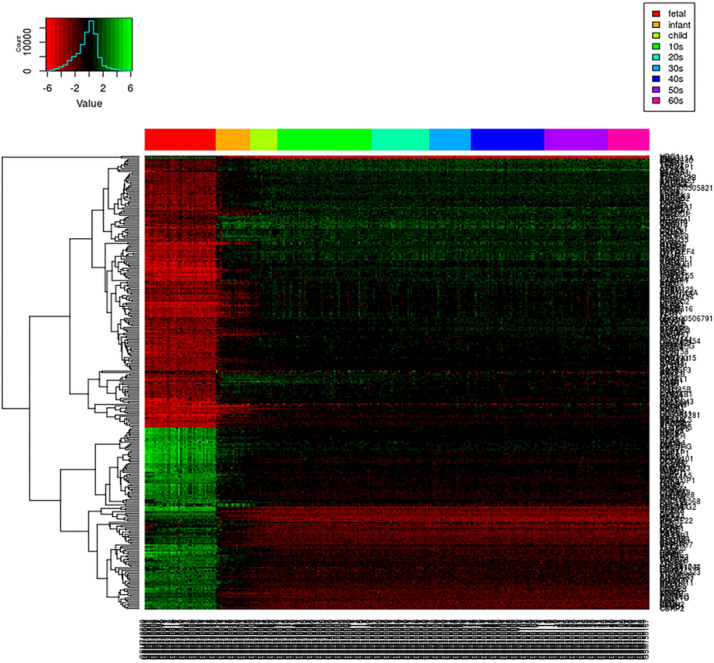
PFC global gene expression changes over time. Each row of the heatmap represents a gene, and each column represents a sample. Each small grid represents the expression of a gene in the sample, with red and green representing relatively low and high expression, respectively. Different colored bars at the top of the graph indicate different age groups to which the sample belong, as shown in the legend on the top right corner.

We first create a gene expression heat map to visualize the age distribution of gene expressions (see Figure 4). A quick glance at the heat map reveals that gene expressions are significantly disturbed (up- or downregulated) in the early stage of the life cycle. From fetus to infant stage, we see a noticeable change in gene expressions. After infant, genes expressions are relatively stable until 60s, and genes clustered together show a relatively smoothed expression pattern. Even from fetus to infant stage, some genes show gradually decreased or increased expressions with gradually fading color in red and green. As the loggle model assumes the smoothness of the graphical topology, the smoothed gene expression patterns across the life span provide empirical evidence to support the loggle model. As a comparison, we also fit the data with the kernel and invar model.

**FIGURE 5:**
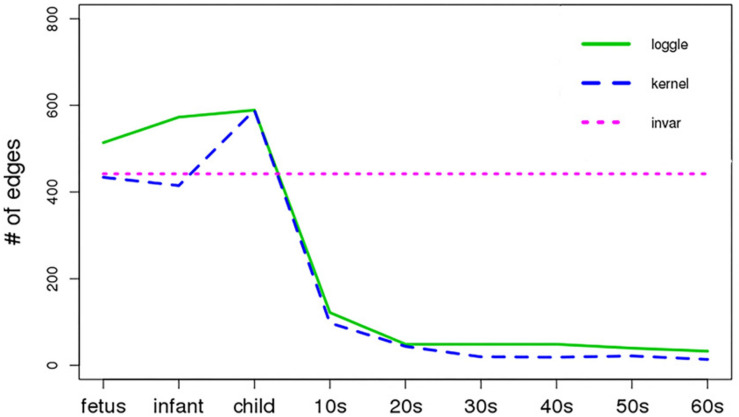
Number of edges vs. age period (*x*-axis). Different line types represent different models.

Figure 5 shows the change in the number of edges of the time-varying network fitted by the three models over time. Table 2 reports the average number of edges and the cross-validation results. We can see that loggle and kernel have fewer average edges, while invar has more edges. In addition, from Figure 5, we can see that the PFC time-varying graph constructed by the invar model does not change throughout the lifespan, so it cannot reflect the changes in the relationship between genes. Since the time-invariant network fitted by the invar model has the same parameters at each time point, no specific CV score can be obtained in this analysis. In contrast, the PFC time-varying graph constructed by the loggle and kernel models captures the developmental pattern over time. The network structure is more complex (e.g., has more edges) during the early stage and then gradually falls into a stationary state, which is consistent with the human prefrontal cortex developmental pattern. Unfortunately, the kernel model has a slight decline in the infant stage, which is contrary to the current understanding of PFC development (the human brain grows at an incomparable rate in the early stages of life) (Liu et al., 2012; Teffer and Semendeferi, 2012). Since the kernel model ignores the smoothness of the graph structure over time, it did not capture the peak of PFC development (early stages of life) when describing the PFC time-varying network. In general, two criteria need to be considered when determining using loggle or kernel: (1) Does the model assumption fit the biological nature of the data well? and (2) Is there a mathematical criterion to determine which method to apply? For (1), loggle appears to better capture the peak of PFC development (child). For (2), the CV score can be used to decide which model fits the data better. In this application, loggle has smaller CV score, hence is preferred than kernel, although the CV score difference is not very significant. In summary, the time-varying graph fitted by the loggle model is more suitable to describe the changes of PFC gene interaction with age, and to identify the peak period of PFC development. Supplementary Table 1 shows the result of the parameter selection of the loggle model at each age stage.

**TABLE 2:** List of average # of edges and cv.score using different method.

Method	Average # of edge	cv.score
loggle	224.2	−309.64
kernel	183.9	−302.77
invar	442.0	–

### Development of Human PFC Time-Varying Network Graph

**FIGURE 6:**
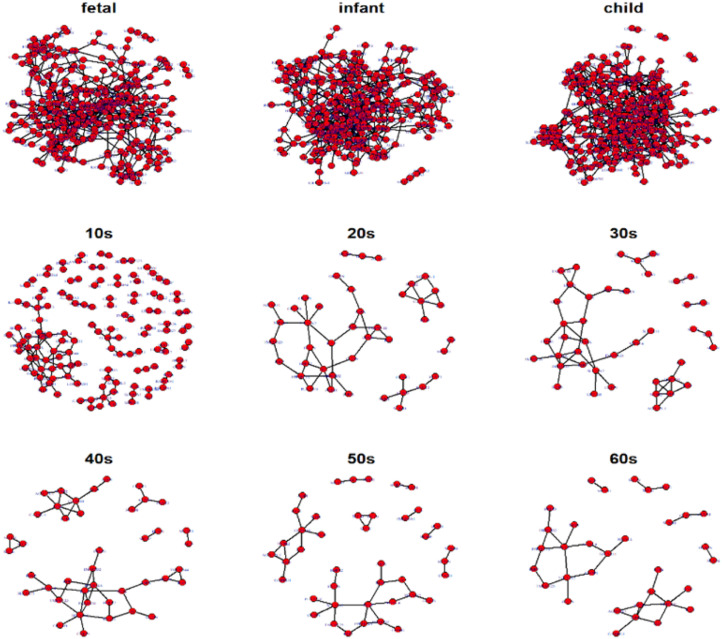
Display of networks corresponding to the nine age periods built with the 300 selected genes. Nodes represent genes and connections between nodes indicate interactions between genes. Genes without connections were removed from the network display for more clear visualization.

**FIGURE 7:**
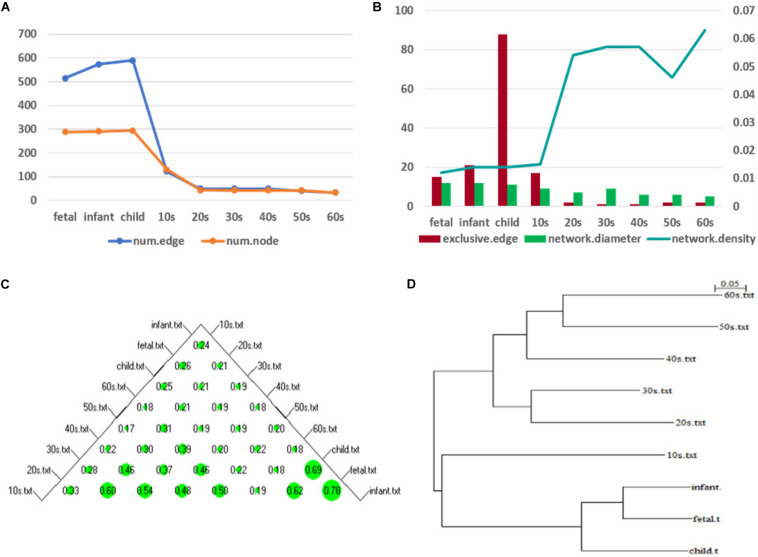
Network topology change **(A)** shows the numbers of edges and nodes included in the network at different ages **(B)** represents the specific edge (left *y*-axis), network diameter (left *y*-axis), and network density (right *y*-axis) in the network corresponding to each age period **(C)** shows a bubble chart of a similarity matrix generated based on the CNSI. Larger bubbles indicate higher similarity of the corresponding two networks **(D)** is a hierarchical clustering tree dendrogram generated based on the similarity matrix.

Hereafter, we focus on the time-varying graph fitted by the loggle model to further analyze the development of the human PFC gene expression network over time. The time-varying graph of the gene interaction network fitted by this model is shown in Figure 6. For the list of edges corresponding to each age group network, please refer to the Supplementary Materials. The trends of change of the number of nodes and the number of edges of the corresponding network are shown in Figure 7A. It is observed that, from the fetus stage to the child stage, with increasing age, the complexity of the PFC gene interaction network begins to increase gradually, peaking in the child stage. During this time, the number of edges of the network is also much higher than the number of nodes (see Figure 7A). Compared with the case at older ages, the inner edge of the network is more complicated. This demonstrates that most of the genes in the PFC region are very active and complexly regulated during this time period, in which promotes the rapid development of PFC. After that, the number of edges decreases rapidly between 10 and 20 years old (child stage to the 10s), and the number of active nodes also decreases. This shows that the development rate of PFC gradually slows down. After the 10s, the numbers of edges and nodes gradually plateau, and the edge composition inside the network is simplified. This indicates that the development speed gradually decreases. It tends to be stable after the 20s. Moreover, after the 50s, it breaks the stability and the number of edges and nodes in the network show a slight downward trend again (Supplementary Table 1). Thus, the development of the PFC time-varying network model presents a cubic trend, with rapid development in early life, a trend of moderate growth in middle age, and then a slight decline later in life. This proves the continuous long-term changes of the PFC gene expression network, echoing the findings of a previous report (Teffer and Semendeferi, 2012).

To illustrate the changes in the network topology in more detail, we calculated several global network properties. As seen in Figure 7B, in the early stage of life (fetus to the 10s), the network diameter is large and the network density is low. After that, the network diameter gradually decreases. Interestingly, the network density then gradually increases in the 10s, although there are slight fluctuations. This implies that the time-varying network graph of PFC is relatively sparse in early life and gradually becomes denser with aging, resulting in a reduction in the robustness of the network. In addition, compared with the late period, there are more exclusive edges in the first four stages of life, and the number of exclusive edges in the child period is the highest, which further indicates that this is a period of rapid development of human PFC. During this period, some unique biological processes occur to promote the development of PFC.

We further analyzed the similarity between the networks of the nine age periods using the CNSI indicator (see section “Materials and Methods). As shown in Figure 7C, the similarity between the networks corresponding to the two periods of fetus and infant is the highest (0.78), followed by infant and child (0.69). The corresponding hierarchical clustering tree (Figure 7D) aggregates these three periods into one category. A closer look at this bubble chart also reveals that the similarity between the 20s and the 30s is also very high (0.6), and the tree diagram puts them in the same cluster. The similarity among the 40s, 50s, and 60s is also high, and their distances in the tree are also short. In contrast, the similarity between the 10s and other networks is low, and the dendrogram places 10s in separate clusters.

Based on these results and previous studies (Teffer and Semendeferi, 2012; Molnár et al., 2019), we divided the development of PFC into three stages, namely, fast development period (fetus, infant, child), deceleration to stationary period (10s, 20s, 30s), and recession period (40s, 50s, 60s). Central analysis was performed separately to identify hub genes at these three different stages.

### Hub Genes Accompanying the Development of Human PFC

**FIGURE 8:**
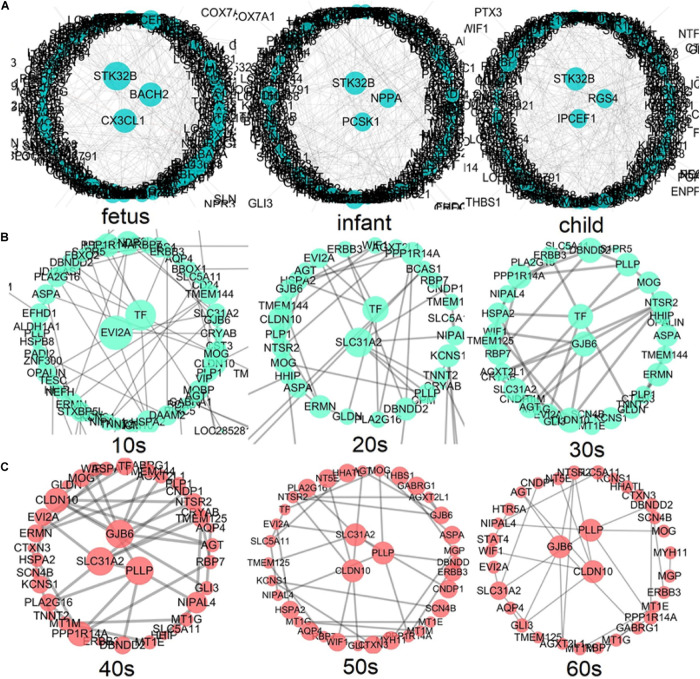
Identification of hub genes throughout the lifespan in PFC development **(A)** shows the fast development period of PFC evolution, namely, fetus, infant, and child; **(B)** shows that the evolution of PFC gradually declined to a stable period, that is, 10s, 20s, and 30s; **(C)** demonstrates the destructive recession period in PFC evolution, namely, 40s, 50s, and 60s. A larger node size in the graph corresponds to a higher node degree. Nodes with the highest degree are considered to be hubs and are placed in the network center.

According to the above development analysis of the network, three developmental stages were considered to identify hub genes (Figure 8). From Figure 8A, we can see that the hub genes in the fast development period of PFC are as follows: *STK32B*, *CX3CL1*, and *BACH2* in the fetus stage; *STK32B*, *PCSK1*, and *NPPA* in infant; and *IPCEF1*, *STK32B*, and *RGS4* in child. The hub genes in the deceleration to stationary period of PFC development (Figure 8B) are as follows: *EVI2A* and *TF* in the 10s, *SLC31A2* and *TF* in the 20s, and *GJB6* and *TF* in the 30s. Finally, the hub genes in the recession period of PFC development (Figure 8C) are as follows: *SLC31A2*, *GJB6*, and *PLLP* in the 40s; *SLC31A2*, *CLDN10*, and *PLLP* in the 50s; and *CLDN10*, *GJB6*, and *PLLP* in the 60s. Interestingly, we found that some genes are consistently identified as hub genes at different age periods within the same stage. For example, the gene *STK32B* is consistently identified as a hub gene at the three age periods during the fast development stage, the gene *TF* functions consistently as a hub gene in the deceleration to stationary period, and the same applies for the gene *PLLP* in the recession period. This indicates the importance of these genes in the development of the human PFC gene network. The biological functions associated with most of them provide further detailed information for discovering biological functions involved in PFC development with age.

We further extracted the subnetworks involving the hub genes at different developmental stages (see Supplementary Figure 1). As shown in the figure, it is clear that within each of the three developmental periods, the subnetworks connecting the hub genes are largely preserved, showing homogeneity within each developmental stage, while subnetworks show large heterogeneity between different stages. This implies that the difference in brain function at different developmental stages may be due to the difference in network connectivity, supporting the importance of network connectivity in brain development revealed by Oldham et al. (2006).

### Development of Five Known Pathways in PFC and the Identification of Hub Genes

**FIGURE 9:**
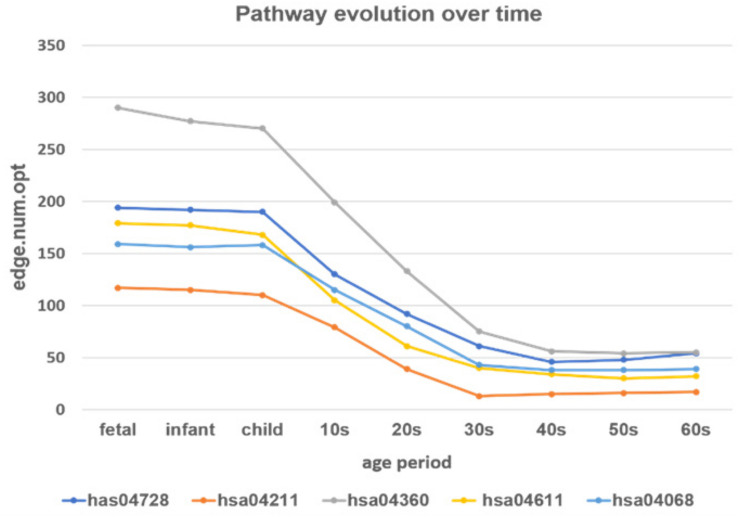
The development of the time-varying networks over different age periods. The y-axis shows the number of edges at each developmental stage fitted by the loggle model.

Here, we selected five pathways related to brain function (see Table 1 for details) to see the developmental trend in PFC over time. The parameter selection results by loggle are shown in Supplementary Table 2. Using the parameters, we constructed network graphs and the network development trends are shown in Figure 9.

From Figure 9 we can see that, as age increases, the number of network edges in these five pathways gradually decreases. From the fetus to the child stage, the network size of these pathways remains largely stable, meaning that most genes involved in these pathways are mostly active in the PFC region during this time period. Then, from the 10s stage to the 30s, the numbers of edges and genes in all pathways gradually decrease; thus, development gradually slows down and eventually remains relatively stable after the 30s. Among the pathways, the pathway hsa04360 (*Axon guidance pathway*) has the most nodes and edges, with the fastest change from child to 30s. Among the five pathways, the *Axon guidance pathway* is most sensitive to age changes, followed by three pathways: the *Dopaminergic synapse pathway* (hsa04728), the *Platelet activation pathway* (hsa04611), and the *FoxO signaling pathway* (hsa04068). These three pathways not only have similar numbers of nodes and edges in PFC, but also the rates of change in the declining period are similar, and the numbers of edges in the stationary period are also similar. In contrast, the *Longevity regulating pathway* (hsa04211) has the fewest nodes and edges, the slowest rate of change in the declining period, and the lowest number of edges in the final stationary stage. The results provide evidence that these pathways are highly active during the fast development stage and are abolished with the slowing down of PFC development.

**FIGURE 10:**
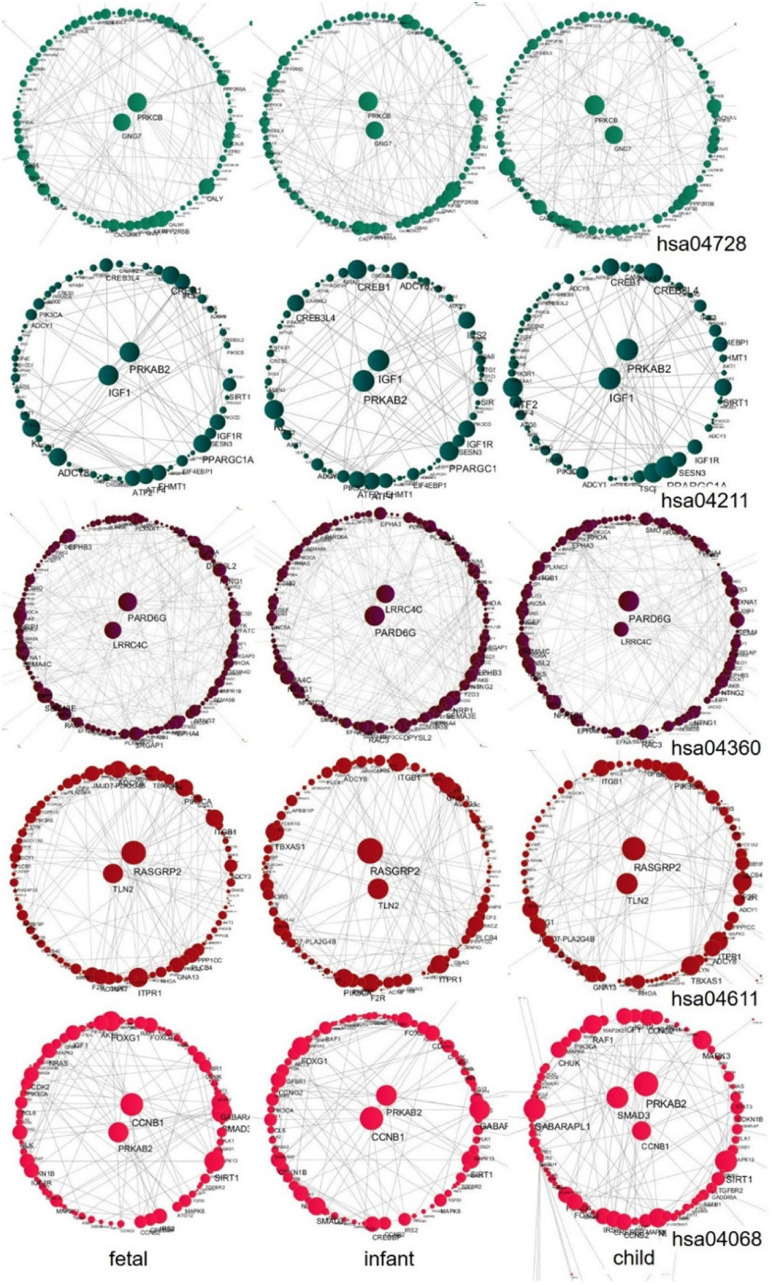
Central analysis of networks corresponding to the fast development stage (fetus, infant, and child) for the five pathways. A larger node corresponds to a larger node degree. Hub genes with large node degree values in each network are placed in the center of the network.

We further explored the hub genes of these pathways at the fast development period (fetus, infant, and child), as shown in Figure 10. We found that the hub genes of each pathway during this period are nearly unchanged. Among them, the hub genes for the *Dopaminergic synapse pathway* (hsa04728) are *PRKCB* and *GNG7*; for the *Longevity regulating pathway* (hsa04211) are *IGF1* and *PRKAB2*; for the *Axon guidance pathway* (hsa04360) are *LRRC4C* and *PARD6G*; for the *Platelet activation pathway* (hsa04611) are *TLN2* and *RASGRP2*; and for the *FoxO signaling pathway* (hsa04068) are *CCNB1* and *PRKAB2*, while one additional gene *SMAD3* is shown in the child stage. We also performed a central analysis of the other two developmental stages, the declining stage (10s, 20s, and 30s) and the stable stage (40s, 50s, and 60s). Owing to limits of space, we placed the results in Supplementary Material. Please see Supplementary Figures 2, 3 for more details.

## Discussion

In this work, to decipher the dynamic temporal development trajectory of PFC region in human brain, we conducted a comprehensive network analysis using transcriptomics data. The loggle model was used to reconstruct the developmental trajectory of the time-varying network graph of gene interaction, and the global network attribute index is used to quantify the network changes. At the same time, the development of PFC was divided into three stages by similarity analysis, and hub genes at different developmental stages were identified. In addition, several known KEGG pathways related to brain function were chosen for analysis to further demonstrate the development trend of PFC.

### The Evolution of Time-Varying Graphs Reveals the Developmental Pattern of Human PFC

Owing to its functional properties, development of the human brain usually continues for a long time, starting with the fetus and continuing through adolescence. PFC is one of the last brain regions to mature (Fuster, 2002; Sushil et al., 2013). Studies have demonstrated from histological and cognitive perspectives that the developmental characteristics of PFC exhibit rapid development in early childhood, decelerate in adolescence, and then gradually reach a mature and stable state in adulthood (Teffer and Semendeferi, 2012), after which a change to a destructive manner occurs in old age (Salthouse, 2009).

Although the macro-level PFC development model has been widely accepted, owing to technological limitations, the trend of development at the gene interaction level has not been clearly described. To fill this gap, in our study, we used time-series gene expression data to construct the developmental pattern of PFC at the molecular level by estimating the time-varying network graphs. We found that, from the fetus to child stages, PFC experiences fast development and most genes are active during this period. This is due to the increase in the number of neurons in the entire cortex as the size of the brain increases and changes in microstructures such as synapses occur throughout childhood (Teffer and Semendeferi, 2012). The density of neurons in the frontal lobe does not peak until later childhood. This period is a critical period for the development of PFC function: 0–6 years old is a critical period for sensory, motor and language development (Marsh et al., 2008); working memory may begin to develop after 8 months (Diamond and Doar, 1989; Fuster, 2001), and cognitive ability related to recognition memory and basic planning skills appears after 5 years old (Hathaway and Newton, 2019); after 6 years old, cognitive development dominates. According to Anderson et al. (2001), the fast development of logical reasoning capabilities that rely on cognitive functions also occurs between the ages of 6 and 9. Thus, training during the rapid development of PFC is very important for future mental development. After that, the development speed drops dramatically. This period is accompanied by a decrease in the volume of gray matter, a corresponding increase in the volume of white matter, and a decrease in synaptic density (Masliah et al., 1993). The frontal executive function may continue to develop during this period and continue into adulthood (Grafman, 1994). After the age of 20, it tends to be stable, and its development is basically maintained at a low level. After the age of 50, a destructive decline occurs, which may be due to a decrease in brain capacity, loss of synapses, and a decline in cognitive ability (Salthouse, 2009). Unsurprisingly, the trend of PFC development coincides with the cubic trajectory mentioned by Lenroot and Giedd (2006). The development of PFC shows continuous and long-term changes throughout the lifespan. Many studies have reported that PFC is most sensitive to changes in aging (Jernigan et al., 2001), which may be closely related to the function of this area, such as in age-related learning, working memory, and other cognitive functions (Salat et al., 1999; Veluw et al., 2012).

### Hub Genes at Different Stages Reflect the Development of Different Functions of PFC

We attempted to elucidate the mechanism driving the development of PFC by performing a central analysis in three stages based on developmental trends; as a result, we identified several hub genes. Hub genes involved in the development of PFC during its fast development phrase are as follows: *STK32B*, *CX3CL1*, *BACH2*, *PCSK1*, *NPPA*, *IPCEF1*, and *RGS4*. Several of these hub genes play specific roles in neuronal differentiation or synapse formation. For example, the gene *STK32B* is upregulated during the fetus period and then enters a suppressed state. This gene encodes a protein involved in synaptic plasticity, learning, memory, and neurodegeneration, and is a key factor in the transmission of information between cells (Temtamy et al., 2008). Therefore, the upregulation of *STK32B* may be related to the development of early cognitive function and synapse formation in PFC. This is not the first time this gene has been found to be expressed in the human cortex (Ciuculete et al., 2018). The genes *CX3CL1* and *PCSK1* are involved in the formation of synapses, with *CX3CL1* encoding a chemokine that is highly expressed in dendritic cells (Gunner et al., 2019), which is upregulated after birth and participates in the development of PFC neurons and synapses. The discovery of this gene during PFC development overlaps with the findings of a previous study (Wruck and Adjaye, 2020). The gene *IPCEF1* is expressed in the brain and reported to be involved in nerve injury-induced changes in membrane receptor trafficking (Guan et al., 2009). This gene has been repeatedly reported in human brain tissue (Sunkin et al., 2012). In this data analysis, we found that this gene is downregulated in the early stage of PFC development and its expression gradually increases with aging. The gene *RGS4* has been shown to be involved in neuron differentiation and neurite growth (Pallaki et al., 2017). An animal test by Huang et al. (Huang et al., 2018) showed that *RGS4* deficiency in the prefrontal cortex may be related to schizophrenia-related behaviors. In short, our analysis results indicate that these genes play a key role in the formation of synapses and other microstructures during the critical period of PFC development, thereby affecting the development of human cognitive functions such as storage, planning or execution.

The hub genes involved in the slowing down to a stable period of PFC development are as follows: *EVI2A*, *SLC31A2*, *TF*, and *GJB6*. These genes are related to the conduction of nerve impulses and the electrophysiological balance of cell membranes. The function of the *EVI2A* gene is related to transmembrane signaling receptor activity. Mladinov et al. (2016) reported that this gene is differentially expressed in the dorsolateral and medial orbitofrontal cortex of patients with schizophrenia. The other three genes are involved in the transmembrane transport of different substances. Among them, the protein encoded by the gene *TF* mediates the transport of iron ions, balances iron levels in the body, and participates in myelination and remyelination of the central nervous system (Carden et al., 2019). It has been shown that dysfunction of this gene is related to Parkinson’s disease (Si et al., 2018). Finally, *GJB6* is regulated by glucocorticoids in the brain to provide energy and maintain the supply of nutrients to the brain (Lu et al., 2018).

The hub genes involved in the late stage of PFC are as follows: *SLC31A2*, *GJB6*, *PLLP*, and *CLDN10*. These genes play active roles in maintaining the structural integrity of the nerve system during PFC decline. For example, gene *PLLP* has been shown to encode myelin structural proteins, and to be involved in the development of myelin around neurons and maintaining the integrity of the myelin structure (Yaffe et al., 2015). Hamacher et al. (2001) reported the isolation of *PLLP* in mammalian brain, and noted that the mutated product of this gene may be involved in Bardet–Biedl syndrome type 2 (BBS2).

By exploring the expected interactions between hub genes and other genes in each subnetwork, we found that although hub genes at different age periods are changing, the subnetworks connecting hub genes within the same developmental period are largely preserved (see Supplementary Figure 1). There is large homogeneity of network connectivity within each of the three developmental periods, but large heterogeneity across different developmental stages. The genes that directly interact with *STK32B* are involved in a variety of tissue differentiation and developmental functions and nerve conduction, for example, regulating cell development and differentiation (*CSRP2*) (Wang et al., 2017), involved in the excitability of neurons in spinal cord and brain tissue (*GLRA2*) (Wegner et al., 2012), extracellular matrix remodeling and Migration (*CCBE1*) (Mesci et al., 2017), regulation of neuronal migration (*SRGAP1*) (Kutys and Yamada, 2014), regulation of signaling pathways involved in development and cell growth (*RSPO3*) (Chen et al., 2018), and axon growth (*C1orf187*) (Riyadh et al., 2014). The genes that directly interact with *TF* are involved in cell-to-cell communication and neuro-related diseases, such as the completion of myelin sheath and the maintenance of cell-to-cell communication (*MOG*) (Tea et al., 2019), neuregulin (*ERBB3*) (Kiavue et al., 2020), participating in schizophrenia (*EVI2A*) (Mladinov et al., 2016), and promote the formation of nodules in the peripheral nervous system (*GLDN*) (Maluenda et al., 2016). The genes that directly interact with *PLLP* are involved in cell-to-cell communication and neurological-related diseases, such as the completion and maintenance of myelin sheath (*MOG*), the ion transporter (*SLC31A2*) (Schweigel-Röntgen, 2014), and Parkinson’s disease (*DBNDD2*) (Kim et al., 2006).

Most of the genes that are pivotal at different stages of PFC development related to brain function development and related diseases, have been evaluated in previous studies; but for some of them there is no clear connection to human brain development. This requires further experimental analysis, although this is beyond the scope of the present study. In summary, our analysis shows that, during the fast development period of PFC, the hub genes mainly regulate the proliferation and differentiation of neurons and the development of synapses. In the stable period of PFC development, the hub genes mainly maintain the stability of PFC in the human brain by maintaining nerve impulses and electrophysiological balance. Finally, during the stage of decline of PFC, the hub genes mainly function to combat the degradation of nerve fibers. This also illustrates the microstructure involved in the development of PFC throughout the lifespan.

### Pathways Regulate PFC Development Through Hub Genes

The five chosen pathways experience changes along with the development of human PFC. Among them, the *Axon guidance pathway* is the most sensitive to aging throughout the lifespan. It is well known that axons are an important component of neurons and play an important role in the development of the human cerebral cortex. During the critical period of human cerebral cortex development, the *Axon guidance pathway* is highly developed. As the number of neurons increases until the PFC develops completely, with aging, the synapses in the frontal lobe begin to be pruned and decline (Masliah et al., 1993), nerve function declines, and synaptic incapacitation occurs. This overlaps with the results of another study describing that synapse-related pathways decline with age (Wruck and Adjaye, 2020). As a pathway directly related to PFC development, this pathway may regulate the development of PFC mainly through the *LRRC4C* and *PARD6G* genes (Figure 10). The gene *LRRC4C* has been reported to be involved in the regulation of axon development and synaptic development, and its deficit can cause neurodevelopmental disorders (Maussion et al., 2016). In the case of the gene *PARD6G*, it was found to be involved in synaptic modification (Marques et al., 2016).

For the *Dopaminergic synapse pathway*, *Platelet activation pathway*, and *FoxO signaling pathway*. As a neuromodulator, dopamine (DA) plays a vital role in the normal cognitive processes of PFC (Seamans and Yang, 2004). The hub genes *PRKCB* and *GNG7* may play important roles in this pathway. The protein encoded by the *PRKCB* gene is involved in a variety of cellular signaling pathways, and it was found in mouse experiments that this kinase may also be involved in regular neuronal function and endocrine regulation, which are related to emotional response behavior (Hayashi et al., 2017). Regarding the hub gene *GNG7*, it was found to be involved in motor control between dopamine-mediated striatum neurons (Sasaki et al., 2013). The brain is one of the regions of the body with an abundance of blood. It is thus unsurprising that the *Platelet activation pathway* develops in parallel with PFC. Our analysis showed the roles of the hub genes *TLN2* and *RASGRP2* in linking this pathway with PFC development. The hub gene *TLN2* is thought to be involved in atherosclerosis (von Essen et al., 2016). The *RASGRP2* gene encodes the main signaling molecule in platelets, and mutations in this gene affect thrombus formation and cause severe bleeding (Canault et al., 2014). The forkhead box O (*FOXO*) transcription factor provides protection for nerve cells during oxidative stress (Maiese et al., 2007). Our analysis revealed that the hub genes *PRKAB2*, *SMAD3*, and *CCNB1* play key roles in the regulation of PFC development in the *FoxO signaling pathway*. The three genes are mainly involved in the positive regulation of AMPK activity (Nagy et al., 2018), signal transduction (Ma et al., 2019), and cell cycle control (Fang et al., 2014).

The *Longevity regulated pathway* showed the slowest pattern of change among the five pathways. The hub genes involved in the *Longevity regulated pathway* are *IGF1* and *PRKAB2*. The proteins encoded by these two genes are involved in the caloric restriction (CR) pathway (Barzilai et al., 2012) and the positive regulation of AMPK activity (Nagy et al., 2018), establishing the connection between the pathway and PFC development.

Our research combines the time-varying networks constructed by the loggle model with traditional network analysis (e.g., similarity analysis, centrality analysis), revealing the characteristics of normal human brain development patterns, and expanding our knowledge of the spatio-temporal event in human brain development. However, the current study does have some limitations. Due to the computational limitation of the algorithm, we can only select a limited number of genes for analysis. The algorithm needs to be further improved to include more gene data. Owing to the specificity of the tissue site, the current gene level analysis of the human cerebral cortex is almost entirely dependent on the examination of post-mortem tissue and the data are not from a cohort study. In addition, we hope to integrate other omics data such as miRNA, DNA methylation, and proteomics data into the network analysis, to obtain a more comprehensive picture of PFC development. We plan to pursue these issues in future work.

## Data Availability Statement

Publicly available datasets were analyzed in this study. This data can be found here: https://www.ncbi.nlm.nih.gov/geo/query/acc.cgi?acc=GSE30272.

## Author Contributions

HW performed the analysis and drafted the manuscript. YW, RF, JS, and ZL participated in data analysis and interpretation. HC and YC conceptualized the idea and revised the manuscript. All authors read and approved the final manuscript.

## Conflict of Interest

The authors declare that the research was conducted in the absence of any commercial or financial relationships that could be construed as a potential conflict of interest.

## Footnotes

1 http://www.ncbi.nlm.nih.gov/geo

## Abbreviations

PFC: Prefrontal cortex
loggle: Local Group Graphical Lasso Estimation
KEGG: Kyoto Encyclopedia of Genes and Genomes
GEO: Gene Expression Omnibus
BBS2: Bardet-Biedl syndrome type 2
FOXO: forkhead box O
CR: caloric restriction
CNSI: CompNet neighbor similarity index
CV: cross-validation.
