# Supplementary material for: Time-Varying Gene Network Analysis of Human Prefrontal Cortex Development
Source: Front Genet. 2020 Nov 16;11:574543. doi: 10.3389/fgene.2020.574543 (PMC7701309; doi:10.3389/fgene.2020.574543)
Supplement: Supplementary file 1 [file Data_Sheet_1.docx]

**Supplemental material for “Time-varying gene network analysis of human prefrontal cortex development”**

Huihui Wang^1^, Yongqing Wu^1^, Ruiling Fang^1^, Jian Sa^1^, Zhi Li^2^, Hongyan Cao^1*^ and Yuehua Cui^3*^

^1^*Division of Health Statistics, School of Public Health, Shanxi Medical University, Taiyuan, Shanxi 030001, PR China*

^2^*Taiyuan Central Hospital of Shanxi Medical University, Department of Hematology, Taiyuan, Shanxi 030001, PR China*

^3^*Department of Statistics and Probability, Michigan State University, East Lansing, MI 48824, USA*

***Correspondence:** Hongyan Cao ([cao_hong_yan@163.com](mailto:cao_hong_yan@163.com)) or Yuehua Cui ([cuiy@msu.edu](mailto:cuiy@msu.edu))

**1. Supplemental tables**

**Table S1**. CV results of the time-varying network., where h.opt, d.opt, λ.opt, and edge.num.opt indicate the optimal *h*, *d*, *λ* and the number of edges selected by CV, respectively.

| **Age period** | **h.opt** | **d.opt** | **λ.opt** | **edge.num.opt** |
| --- | --- | --- | --- | --- |
| fetal | 0.3 | 0.25 | 0.35 | 514 |
| infant | 0.3 | 0.1 | 0.33 | 573 |
| child | 0.3 | 0.01 | 0.29 | 589 |
| 10s | 0.3 | 0.05 | 0.35 | 122 |
| 20s | 0.3 | 0.075 | 0.31 | 49 |
| 30s | 0.3 | 0.15 | 0.31 | 49 |
| 40s | 0.3 | 0.3 | 0.31 | 49 |
| 50s | 0.3 | 0.3 | 0.31 | 40 |
| 60s | 0.3 | 0.25 | 0.33 | 33 |

**Table S2**. CV results of the time-varying network., where h.opt, d.opt, λ.opt, and edge.num.opt indicate the optimal *h*, *d*, *λ* and the number of edges selected by CV, respectively.

| Pathway | Parameter | fetal | infant | child | 10s | 20s | 30s | 40s | 50s | 60s |
| --- | --- | --- | --- | --- | --- | --- | --- | --- | --- | --- |
| hsa04728 | h.opt | 0.3 | 0.3 | 0.3 | 0.3 | 0.3 | 0.3 | 0.3 | 0.3 | 0.3 |
|  | d.opt | 0.3 | 0.3 | 0.3 | 0.3 | 0.3 | 0.3 | 0.3 | 0.3 | 0.3 |
|  | λ.opt | 0.2 | 0.2 | 0.2 | 0.2 | 0.2 | 0.2 | 0.2 | 0.2 | 0.2 |
|  | edge.num.opt | 194 | 192 | 190 | 130 | 92 | 61 | 46 | 48 | 54 |
| hsa04211 | h.opt | 0.3 | 0.3 | 0.3 | 0.3 | 0.3 | 0.3 | 0.3 | 0.3 | 0.3 |
|  | d.opt | 0.3 | 0.3 | 0.15 | 0.3 | 0.3 | 0.25 | 0.3 | 0.3 | 0.3 |
|  | λ.opt | 0.2 | 0.2 | 0.2 | 0.2 | 0.2 | 0.2 | 0.2 | 0.2 | 0.2 |
|  | edge.num.opt | 117 | 115 | 110 | 79 | 39 | 13 | 15 | 16 | 17 |
| hsa04360 | h.opt | 0.3 | 0.3 | 0.3 | 0.3 | 0.3 | 0.3 | 0.3 | 0.3 | 0.3 |
|  | d.opt | 0.3 | 0.3 | 0.3 | 0.3 | 0.3 | 0.3 | 0.3 | 0.3 | 0.3 |
|  | λ.opt | 0.2 | 0.2 | 0.2 | 0.2 | 0.2 | 0.2 | 0.2 | 0.2 | 0.2 |
|  | edge.num.opt | 290 | 277 | 270 | 199 | 133 | 75 | 56 | 54 | 55 |
| hsa04611 | h.opt | 0.3 | 0.3 | 0.3 | 0.3 | 0.3 | 0.3 | 0.3 | 0.3 | 0.3 |
|  | d.opt | 0.3 | 0.3 | 0.3 | 0.3 | 0.3 | 0.3 | 0.3 | 0.3 | 0.3 |
|  | λ.opt | 0.2 | 0.2 | 0.2 | 0.2 | 0.2 | 0.2 | 0.2 | 0.2 | 0.2 |
|  | edge.num.opt | 179 | 177 | 168 | 105 | 61 | 40 | 34 | 30 | 32 |
| hsa04068 | h.opt | 0.3 | 0.3 | 0.3 | 0.3 | 0.3 | 0.3 | 0.3 | 0.3 | 0.3 |
|  | d.opt | 0.3 | 0.3 | 0.05 | 0.3 | 0.3 | 0.3 | 0.3 | 0.3 | 0.3 |
|  | λ.opt | 0.2 | 0.2 | 0.2 | 0.2 | 0.2 | 0.2 | 0.2 | 0.2 | 0.2 |
|  | edge.num.opt | 159 | 156 | 158 | 115 | 80 | 43 | 38 | 38 | 39 |

**2. Supplemental figures**

Fig. S1 shows the extracted subnetworks at three developmental stages involving the bub genes. The three developmental stages are: fast development period (fetus, infant, and child); deceleration to stationary period (10s, 20s, and 30s); and recession period (40s, 50s, and 60s).

Fig. S2 shows the central analysis results corresponding to the 10s, 20s and 30s for the five pathways. The hub genes corresponding to the 10s for pathway hsa04728 are *GRIA4* and *CALY*; for pathway hsa04211 are *IGF1* and *KL*; for pathway hsa04360 are *PARD6G* and *UNC5A*; for pathway hsa04611 are *TLN2* and *GNA13*; for pathway hsa04068 is *CREBBP*. The hub genes corresponding to the 20s for pathway hsa04728 are *CALM3* and *PPP3CB*; for pathway hsa04211 are *CREB3L4* and *ATF2*; for pathway hsa04360 are *PARD6G* and *PARD6A*; for pathway hsa04611 is *TBXAS1*; for pathway hsa04068 are *PRKAB2* and *TGFBR2*. The hub genes corresponding to the 30s for pathway hsa04728 are *CALM3* and *PPP3CB*; for pathway hsa04211 are *ULK1* and *TP53*; for pathway hsa04360 are *ROCK2*, *PPP3CB* and *PPP3CA*; for pathway hsa04611 are *TBXAS1* and *FCGR2A*; for pathway hsa04068 are *MDM2* and *SGK1*.

Fig. S3 shows the central analysis results corresponding to the 40s, 50s and 60s for the five pathways. The hub genes corresponding to the 40s for pathway hsa04728 are *PPP3CB* and *PRKACB*; for pathway hsa04211 is *ULK1*; for pathway hsa04360 are *PPP3CB* and *ROCK2*; for pathway hsa04611 are *TBXAS1*, *FCGR2A* and *GNAI2*; for pathway hsa04068 are *GABARAP*, *S1PR1* and *PIK3CB*. The hub genes corresponding to the 50s for pathway hsa04728 are *PPP3CB* and *PRKACB*; for pathway hsa04211 is *ULK1*; for pathway hsa04360 is *PPP3CB*; for pathway hsa04611 are *FCER1G*, *TBXAS1* and *FCGR2A*; for pathway hsa04068 is *GABARAP*. The hub genes corresponding to the 60s for pathway hsa04728 are *PRKACB* and *PPP3CB*; for pathway hsa04211 is *ULK1*; for pathway hsa04360 is *PPP3CB*; for pathway hsa04611 is *FCER1G*; for pathway hsa04068 is *GABARAP*.


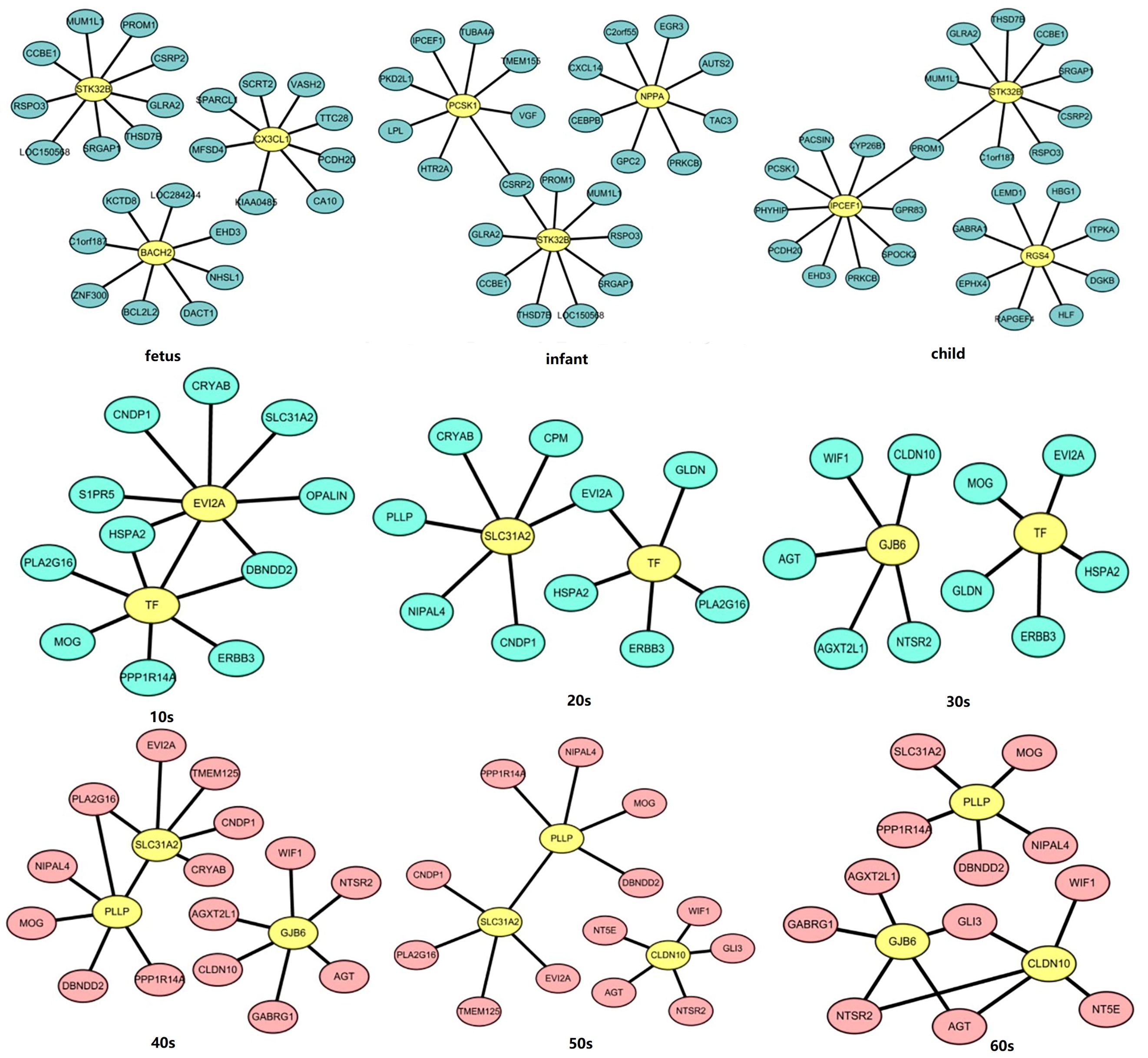


**Fig. S1.** Predicted regulatory relationship between hub genes and other network genes. The yellow nodes represent the hub genes at each stage.

**
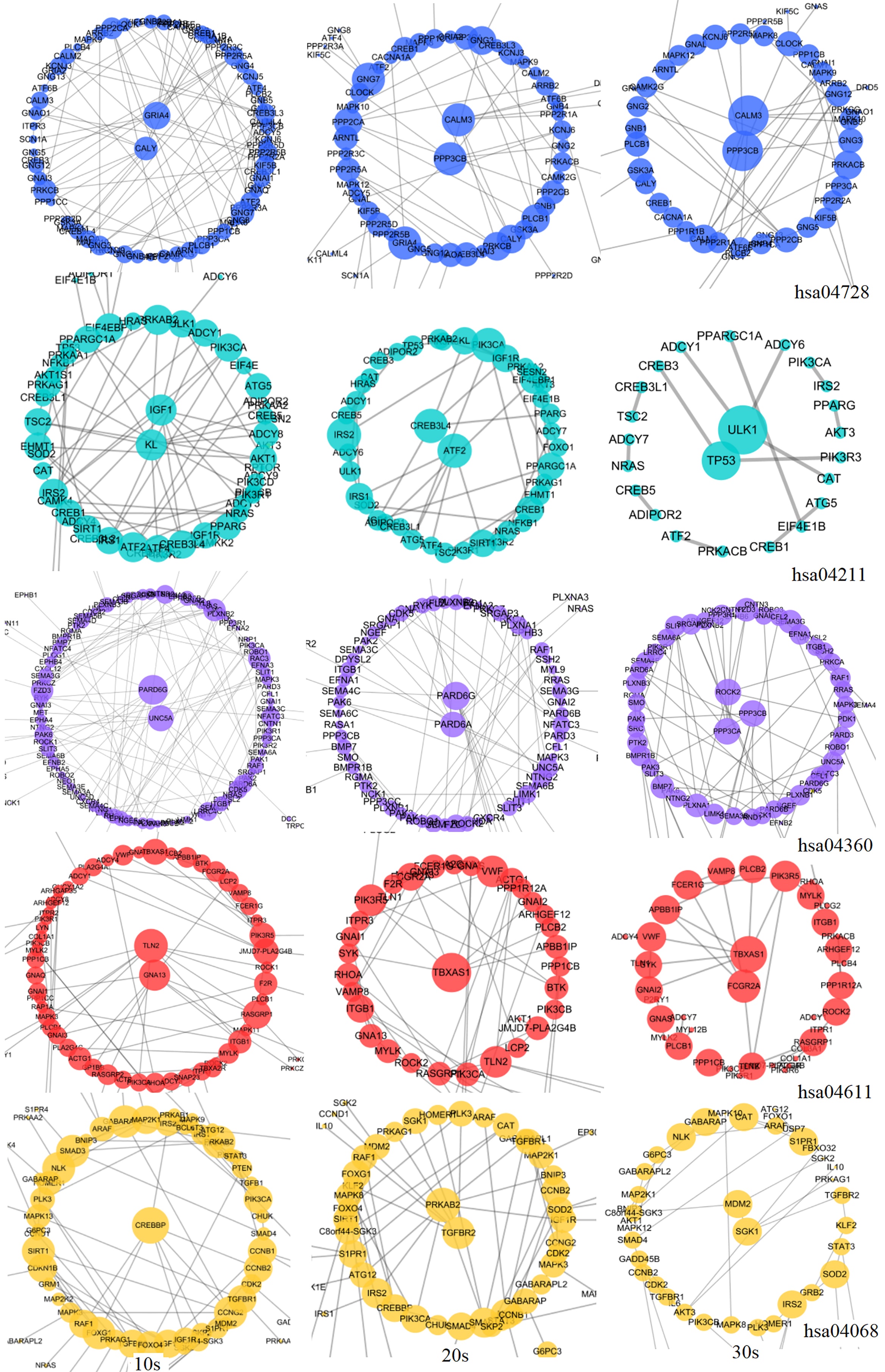
**

**Fig. S2.** Central analysis of networks corresponding to the 10s,20s and 30s for the five pathways. The larger the node, the larger the corresponding node degree. Hub genes with large node degree values in each network are placed in the center of the network.

**
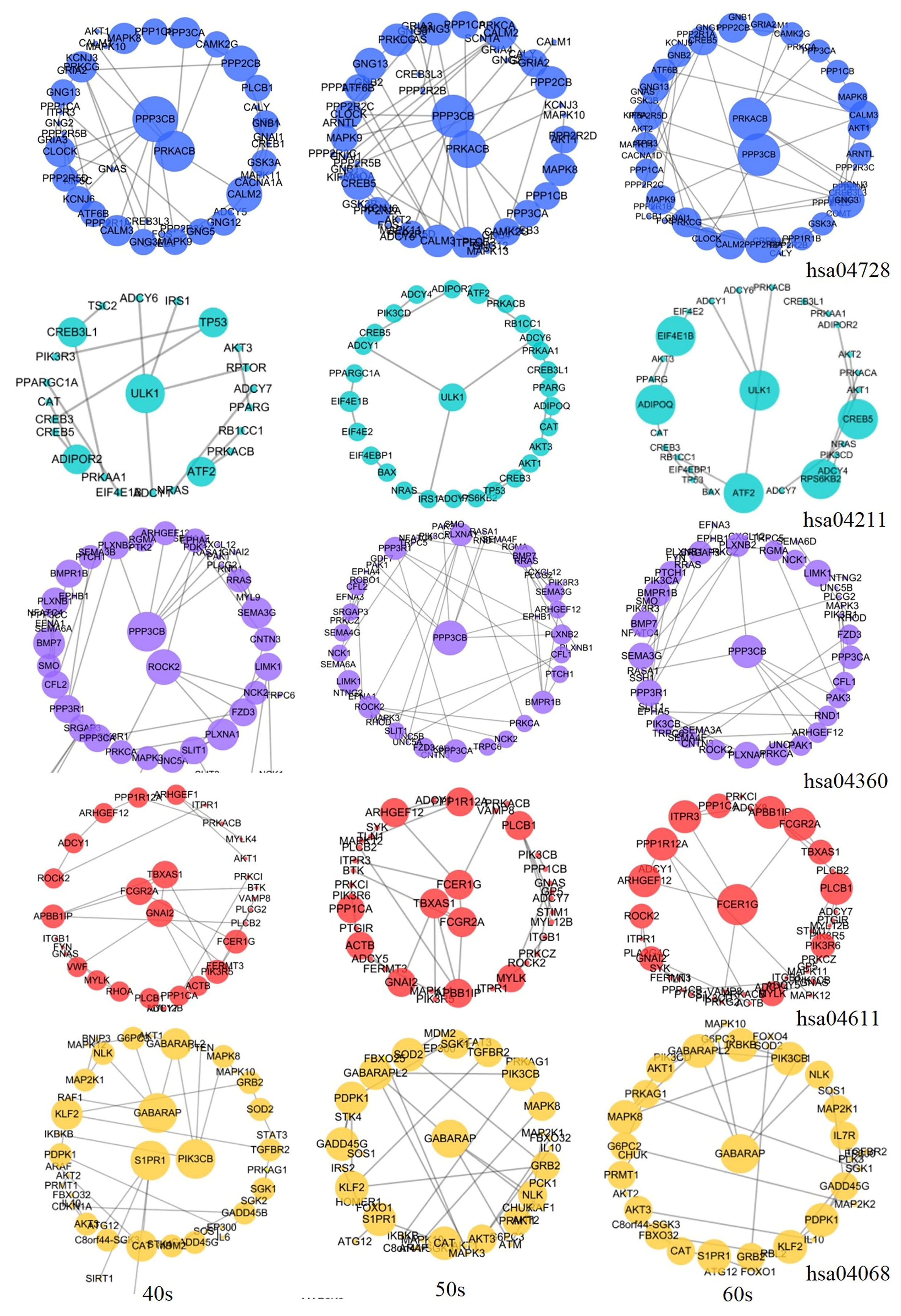
**

**Fig. S3.** Central analysis of networks corresponding to the 40s,50s and 60s for the five pathways. The larger the node, the larger the corresponding node degree. Hub genes with large node degree values in each network are placed in the center of the network.
